# Proteomics of Rice—*Magnaporthe oryzae* Interaction: What Have We Learned So Far?

**DOI:** 10.3389/fpls.2019.01383

**Published:** 2019-10-29

**Authors:** Qingfeng Meng, Ravi Gupta, Cheol Woo Min, Soon Wook Kwon, Yiming Wang, Byoung Il Je, Yu-Jin Kim, Jong-Seong Jeon, Ganesh Kumar Agrawal, Randeep Rakwal, Sun Tae Kim

**Affiliations:** ^1^Department of Plant Bioscience, Pusan National University, Miryang, South Korea; ^2^Department of Botany, School of Chemical and Life Science, Jamia Hamdard, New Delhi, India; ^3^Department of Plant Microbe Interactions, Max-Planck Institute for Plant Breeding Research, Cologne, Germany; ^4^Department of Horticultural Bioscience, Pusan National University, Miryang, South Korea; ^5^Graduate School of Biotechnology and Crop Biotech Institute, Kyung Hee University, Yongin, South Korea; ^6^Research Laboratory for Biotechnology and Biochemistry (RLABB), Kathmandu, Nepal; ^7^GRADE (Global Research Arch for Developing Education) Academy Private Limited, Birgunj, Nepal; ^8^Faculty of Health and Sport Sciences, University of Tsukuba, Tsukuba, Japan

**Keywords:** rice blast disease, plant−pathogen interaction, proteomics, signalling, effectors

## Abstract

Rice blast disease, caused by *Magnaporthe oryzae*, is one of the major constraints to rice production, which feeds half of the world’s population. Proteomic technologies have been used as effective tools in plant−pathogen interactions to study the biological pathways involved in pathogen infection, plant response, and disease progression. Advancements in mass spectrometry (MS) and apoplastic and plasma membrane protein isolation methods facilitated the identification and quantification of subcellular proteomes during plant-pathogen interaction. Proteomic studies conducted during rice−*M. oryzae* interaction have led to the identification of several proteins eminently involved in pathogen perception, signal transduction, and the adjustment of metabolism to prevent plant disease. Some of these proteins include receptor-like kinases (RLKs), mitogen-activated protein kinases (MAPKs), and proteins related to reactive oxygen species (ROS) signaling and scavenging, hormone signaling, photosynthesis, secondary metabolism, protein degradation, and other defense responses. Moreover, post−translational modifications (PTMs), such as phosphoproteomics and ubiquitin proteomics, during rice−*M. oryzae* interaction are also summarized in this review. In essence, proteomic studies carried out to date delineated the molecular mechanisms underlying rice-*M. oryzae* interactions and provided candidate proteins for the breeding of rice blast resistant cultivars.

## Introduction

Food security is becoming a global issue, especially for staple crops such as rice, which has driven an increased focus on developing and improving approaches for crop protection. Rice (*Oryza sativa*) is the primary staple food crop for over 50% of the world’s population. Throughout the growing season, a variety of pathogens, including fungi, bacteria, viruses, and nematodes, infect rice plants and cause significant yield losses. Rice blast disease is a major factor influencing stable rice production in many rice-growing countries around the globe. The disease is caused by the fungal pathogen *Magnaporthe oryzae*, which was differentiated from *Magnaporthe grisea* based on phylogenetic analysis and inter-strain fertility testing (Couch and Kohn, 2002).

*M. oryzae* is a hemibiotroph pathogen, which establishes a biotrophic interaction with the host at the early infection stages. The infection cycle of *M. oryzae* begins at the rice leaf surface with a three-celled conidium. The spore attaches to the hydrophobic surface of the rice leaf and produces a germ tube, followed by flattening and hooking at its tip before differentiating into an appressorium. The three-celled conidium undergoes autophagy cell death, while the single-celled appressorium matures and generates enormous turgor, which translates into a mechanical force to puncture the leaf cuticle *via* formation of a narrow penetration peg at the base of the appressorium. The penetrated hyphae breach the epidermal cell wall and invade the epidermal cells. Subsequently, the hyphae invade neighboring cells to spread the biotrophic infection, and initially infected cells enter a necrotrophic phase (Ebbole, 2007). Fungal hyphae have been shown to grow exclusively in first-infected cells, and then invade the neighboring cells at 24 h post-infection (hpi) and 36 hpi, respectively (Cao et al., 2016). The rice blast disease lesions emerge between 72 hpi and 96 hpi and a substantial number of new spores are generated from aerial conidiophore under humid conditions (Wilson and Talbot, 2009).

The most effective and environmentally conscious approach to control this deadly disease is the breeding of resistant crops (Dodds and Rathjen, 2010). For a successful infection, the pathogen must breach the plant’s basic, passive defense mechanisms, including physical barriers (e.g., cuticle, cell wall), and constitutively generated anti-microbial compounds to obtain nutrients from the plant and complete its lifecycle. To promote pathogenesis, the pathogen delivers secreted proteins (Vir proteins or Avr effectors) or other small molecules (e.g., lipids, nucleic acids, carbohydrates) into the host cells that function to compromise the host’s immunity (Gupta et al., 2015a).

To win the arms race, plants have evolved an innate, two-layered immune system, commonly referred to as a zig-zag model (Jones and Dangl, 2006). The first line of plant immunity is initiated from the perception of conserved extracellular pathogen-associated molecular patterns (PAMPs) by plasma membrane (PM)-localized pattern recognition receptors (PRRs), and is known as PAMP-triggered immunity (PTI; Boller and Felix, 2009). PTI is relatively weaker; however, in many cases, it is sufficient to defend against the pathogen’s attack and protect plants from disease. To overcome PTI, pathogens secrete effector proteins into the host cells, resulting in effector-triggered susceptibility (ETS). However, the resistance (R)-gene products of plants can directly or indirectly recognize the pathogen’s secreted effectors to activate a second line of defense known as effector-triggered immunity (ETI), culminating in hypersensitive responses (HRs; Cui et al., 2015).

It was shown in Arabidopsis that PTI and ETI share most downstream signaling components, such as MAPK cascades, callose deposition, calcium signal activation, and dominated signaling sectors (Tsuda and Katagiri, 2010; Dong et al., 2015). However, the stratagems of using those components differ between PTI and ETI. PTI causes transient signaling, whereas ETI triggers more prolonged and robust responses (Tsuda et al., 2013), resulting in different immune outputs for pathogen resistance in local and systemic tissues.

During a pathogen attack, phytohormones including salicylic acid (SA), jasmonic acid (JA), and ethylene (ET), play key roles in mediating plant immunity. SA activates systemic acquired resistance (SAR) in Arabidopsis which is primarily mediated by a transcriptional cofactor non-expressor of pathogenesis-related genes-1 (NPR1) (Dong, 2004). A similar NPR1 mediated SA signaling pathway has been identified in rice. However, rice resistance against *M. oryzae* appears mediated by a WRKY45-dependent pathway downstream of SA (Shimono et al., 2007). Results reported to date suggest that SA signaling is bifurcated into NPR1-dependent and WRKY45-dependent pathways in rice (Shimono et al., 2007; Jiang et al., 2010). In dicots, including Arabidopsis, SA regulates immunity against biotrophic pathogens, while JA regulates stress responses especially against herbivores and necrotrophic pathogens (Browse, 2009). However, this model does not fully fit into the monocots. For instance, rice contains high endogenous SA levels, and SA levels are not increased upon infection of compatible and incompatible types of *M. oryzae* (Silverman et al., 1995). Further experiments suggest that JA signaling is activated upon infection of biotrophic bacterial pathogen *Xanthomonas oryzae* pv *oryzae* and hemi-biotrophic fungal pathogen *M. oryzae* (Mei et al., 2006; Jiang et al., 2010; Yamada et al., 2012), indicating that the JA signaling pathway may play a major role in rice immunity against biotrophic and hemi-biotrophic pathogens, in contrast to Arabidopsis, where it is activated only in response to necrotrophic pathogens. However, more detailed genetic evidence must first be gathered.

Several proteomics studies have been conducted over the past decade to decipher the mechanisms of rice-*M. oryzae* interactions. However, the majority of these studies utilized a two-dimensional gel electrophoresis (2-DE)-based approach, resulting in the identification of fewer distinct proteins. Recent advances in proteomics, especially the commencement of shotgun proteomics, have allowed the identification of a much higher number of proteins as compared to the gel-based proteomics approach (Gupta et al., 2015b). Moreover, utilization of label-free, iTRAQ and TMT-labeled based quantitative proteomics has made the quantification of peptides more automated and reliable. Here, we summarize the major findings of the proteomics studies in the rice-*M. oryzae* pathosystem, providing a global insight into proteome changes in response to *M. oryzae* infection and pathogen elicitor treatments in rice, as well as a new insight into the molecular mechanism of rice resistance against *M. oryzae via* comparisons between compatible and incompatible interactions.

## Immune System in Rice-*M. oryzae* Interaction

The major components involved in rice−*M. oryzae* interactions include resistance R genes and PRRs from rice, as well as effectors and PAMPs from *M. oryzae*. Of these, R genes and effectors are well studied as a famous gene for gene resistance. In contrast, fewer PRRs and PAMPs have been identified to date in rice−*M. oryzae* pathosystem. Several rice PRRs perceive conserved *M. oryzae*-derived PAMPs including chitin, MSP1 (a cerato-platanin protein with four conserved cysteine residues, secreted from *M. oryzae*, which induces PTI in rice), and MoHrip1 (an Alt A 1 [AA1] family protein, secreted from *M. oryzae*, inducing PTI in rice; Kaku et al., 2006; Wang et al., 2016; Zhang et al., 2017). Among these, PRRs involved in the perception of chitin fragments have already directly or indirectly been identified, and include chitin oligosaccharide elicitor-binding protein (CEBiP), chitin elicitor receptor kinase 1 (CERK1), and LysM domain-containing protein 4 (LYP4) and 6 (LYP6; **Table 1**; Kaku et al., 2006; Liu et al., 2012).

**Table 1:** Rice PRRs and cognate *M. oryzae* PAMPs.

Resistance proteins	*M. oryzae* PAMPs	Rice PRRs	PRR types	References
PRRs	Chitins	CEBiP	LysM-RLP	(Kaku et al., 2006)
		CERK1	LysM-RLK	(Shimizu et al., 2010)
		LYP4	LysM-RLP	(Liu et al., 2012)
		LYP6	LysM-RLP	(Liu et al., 2012)
	MSP1	Unknown	Unknown	(Wang et al., 2016)
	MoHrip1	Unknown	Unknown	(Zhang et al., 2017)
	Unknown	Pi-d2	β-lectin-RLK	(Xuewei et al., 2006)

**Figure 1:**
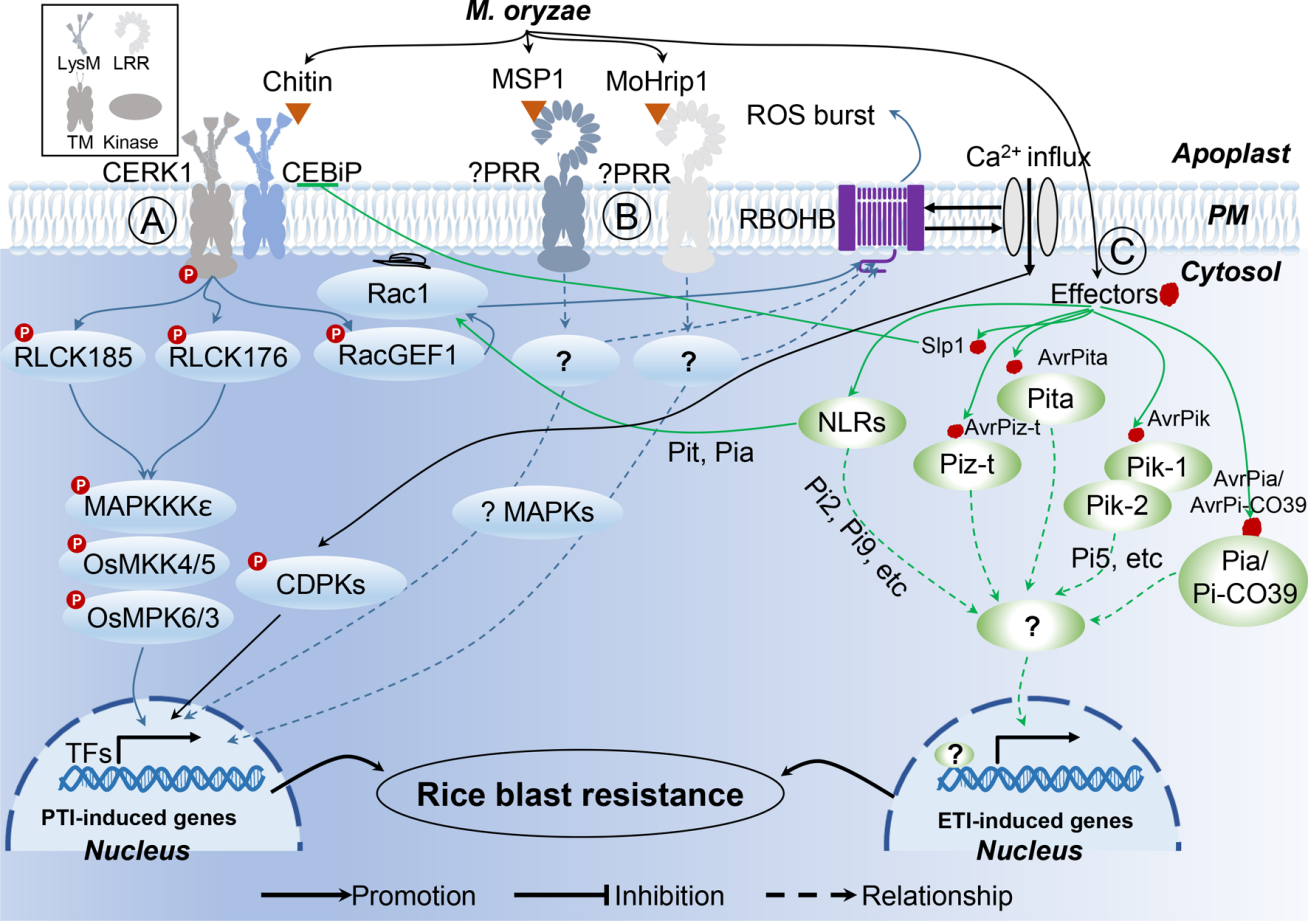
Rice innate immunity signaling pathways triggered by *M. oryzae*. **(A)** Two major rice receptor-like kinase (RLK) pattern recognition receptor (PRR) proteins, CERK1 and CEBiP, perceive the pathogen-activated molecular patterns (PAMPs) chitin, to trigger a rice PAMP-triggered immunity (PTI). **(B)** Unknown PRRs recognize PAMP MSP1 and MoHrip1, respectively. **(C)** In rice-*M. oryzae* interactions, recognition models between *Avr* effectors and R proteins are characterized.

After formation of the PRR complex with chitin and CEBiP, CERK1 phosphorylates the guanine nucleotide exchange factor RacGEF1, which, in turn, activates the small GTPase OsRac1 (**Figure 1**; Akamatsu et al., 2013). In addition to OsRacGEF1, RLCK176, and RLCK185, both function downstream of OsCERK1 in chitin- and peptidoglycan-induced plant immunity (Yamaguchi et al., 2013; Ao et al., 2014). The other two PAMPs, MSP1 (Wang et al., 2016) and MoHrip1 (Zhang et al., 2017), both induce blast-resistance in rice; however, the PRRs involved in the recognition of these two PAMPs are yet to be identified. Moreover, the mechanism of MSP1 and MoHrip1-induced PTI responses are also elusive due to limited data, pointing to a need for further exploration to better understand these two recently identified PAMPs.

Owing to the central involvement of R genes in ETI, more than 100 rice R genes conferring resistance to *M. oryzae* have been identified, 27 of which have already been cloned (Liu and Wang, 2016). In the case of *M. oryzae*, a total of 13 Avr effector genes have been cloned (**Table 2**), which have previously been reviewed (Sharma et al., 2012; Liu et al., 2014b). According to the description of Liu et al., several patterns of R−Avr interaction in *M. oryzae* were found (**Figure 1**; Liu et al., 2014b; Liu and Wang, 2016). Pita/AvrPita and Piz-t/AvrPiz-t are examples of recognition of a single Avr gene by a single dominant R gene (Jia et al., 2000). Moreover, a single Avr protein may require two R proteins acting together. For instance, three such NB-LRR-type R-gene pairs (Pik-1 and Pik-2, Pi5-1 and Pi5-2, a locus called Pia or Pi-CO39 consisting of RGA4 and RGA5) have been identified in rice, which confer Pik-, Pi5-, and Pia/Pi-CO39-mediated resistance, respectively. Remarkably, the first protein structure study of CC-type NLR protein ZAR1 indicated that the active complex of ZAR resistosome, which is associated with the PM, leads to cell death initiation and disease resistance, suggesting the direct role of R proteins in pathogen resistance *via* recognition of effectors from pathogens (Wang et al., 2019a; Wang et al., 2019b). These findings provided the possible mechanism of CC-NLR-mediated ETI in plant immunity. However, structural analysis showed that TIR-NBS LRR mediates cell death signaling in response to pathogens through cleavage of the metabolic cofactor nicotinamide adenine dinucleotide (NAD+) (Horsefield et al., 2019; Wan et al., 2019). These findings suggest that R protein mediates plant immune response though multiple mechanisms. Therefore, structure-based analyses of rice R genes involved in *M. oryzae* resistance may prove beneficial for the understanding of plant immune mechanisms.

**Table 2:** Resistance genes in rice and effectors in *M. oryzae*.

Resistance	*M. oryzae* effectors	Rice R proteins	R protein types	References
R proteins	AvrPib	Pib	NB-LRR	(Wang et al., 1999; Zhang et al., 2015)
	AvrPi-ta	Pi-ta	NB-LRR	(Bryan et al., 2000; Orbach et al., 2000)
	AvrPi9	Pi9	NB-LRR	(Qu et al., 2006; Wu et al., 2015)
	Unknown	Pi2	NB-LRR	(Zhou et al., 2006)
	AvrPiz-t	Piz-t	NB-LRR	(Li et al., 2009)
	ACE1	Pi33	Unknown	(Böhnert et al., 2004)
	AvrPii	Pii	Unknown	(Yoshida et al., 2009)
	Unknown	Pi36	NB-LRR	(Liu et al., 2007)
	Unknown	Pi37	NB-LRR	(Lin et al., 2007)
	Avr-Pik/km/kp	Pikm	NB-LRR	(Ashikawa et al., 2008; Yoshida et al., 2009)
	Avr-Pik/km/kp	Pik	NB-LRR	(Zhai et al., 2010)
	Avr-Pik/km/kp	Pikp	NB-LRR	(Yuan et al., 2011)
	Unknown	Pi50	NB-LRR	(Su et al., 2015)
	Unknown	Pi64	NB-LRR	(Ma et al., 2015)
	Unknown	Pit	NB-LRR	(Hayashi and Yoshida, 2008)
	Unknown	Pi5	NB-LRR	(Lee et al., 2009)
	Unknown	Pid3	NB-LRR	(Shang et al., 2009)
	Unknown	Pid3-A4	NB-LRR	(Lv et al., 2013)
	Unknown	Pi54	NB-LRR	(Shang et al., 2009)
	Unknown	Pish	NB-LRR	(Takahashi et al., 2010)
	Avr-Pia	Pia	NB-LRR	(Okuyama et al., 2011)
	Avr1-CO39	Pi-CO39	NB-LRR	(Ribot et al., 2012)
	Unknown	Pi25	NB-LRR	(Chen et al., 2011)
	Unknown	Pi1	NB-LRR	(Hua et al., 2012)
	Unknown	Pb1	NB-LRR	(Hayashi et al., 2010)
	Unknown	Pi66(t)	NB-LRR	(Liang et al., 2016)
	Unknown	Pi65(t)	NB-LRR	(Zheng et al., 2016)
	Unknown	PigmR	NB-LRR	(Deng et al., 2017)
Atypical resistance	PWL2	Unknown	Unknown	(Sweigard et al., 1995)
	Unknown	Pi21	Proline-containing protein	(Fukuoka et al., 2009)
	Unknown	Ptr	Protein with four Armadillo repeats	(Zhao et al., 2018)
	Unknown	BSR-D1	C_2_H_2_-type transcription factor	(Li et al., 2017)
	Unknown	BSR-K1	A TPRs-containing protein	(Zhou et al., 2018)

Pyramiding R genes for disease resistance is a markedly time-consuming process; thus, deployment of genes conferring broad-spectrum, durable resistance is highly favored by breeders. To date, four atypical R genes (Pi21, Ptr, BSR-D1, and BSR-K1), conferring non-race-specific resistance, have been isolated from rice (**Figure 1**, **Table 2**; Fukuoka et al., 2009; Li et al., 2017; Zhao et al., 2018; Zhou et al., 2018), which differ in mechanism with respect to R gene-mediated resistance, such as the adoption of unique motifs and epigenetic regulation. Increased efforts towards the exploitation of broad-spectrum resistance against *M. oryzae* should be undertaken in the near future.

## Key Proteome Responses Underlying Rice Resistance to *M. oryzae*

Rice proteomics research, including biotic and abiotic stresses, has been comprehensively reviewed (Agrawal and Rakwal, 2011; Kim et al., 2014). Proteomics studies of rice have been conducted during rice−*M. oryzae* interaction, either to determine the plant’s responses to pathogen attacks or to understand the mechanisms of plant resistance against pathogens. Most of these studies in rice−*M. oryzae* interactions focus on the comparison between incompatible and compatible interactions, revealing important proteome reorganization involved in rice resistance against *M. oryzae*. The incompatible interaction between plant and pathogen is a dynamic process focusing on pathogen recognition, signal transduction, and metabolic adjustment to prevent disease, in turn, aiming at an enhancement of plant resistance.

**Figure 2:**
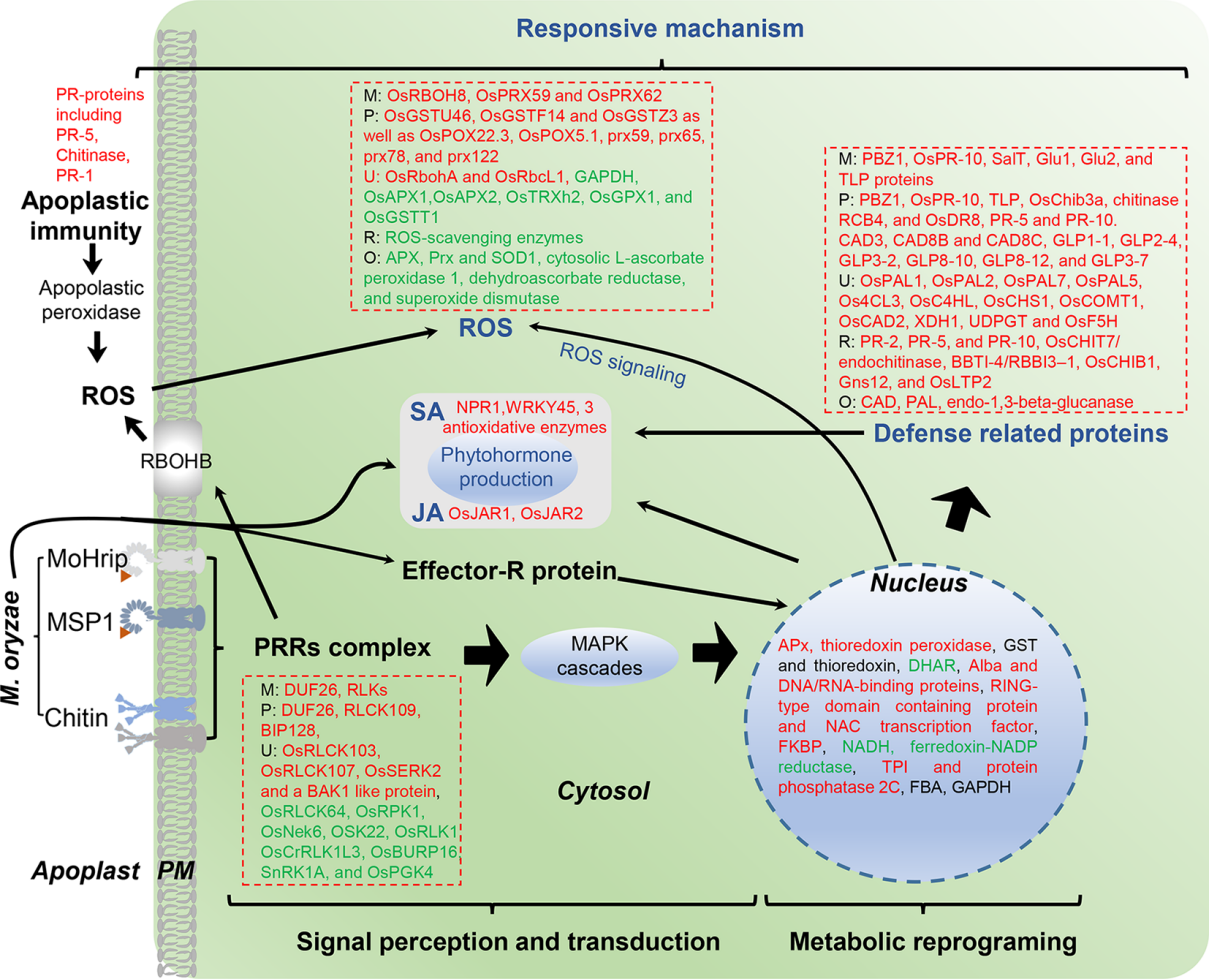
Overview of identified proteins from rice leaves after *M. oryzae* infection. M, *M. oryzae* infection; P, PAMP-induced resistance; R, R protein-mediated resistance; O, Other type of resistance; U, ubiquitination level of proteins. Red color indicates up-regulated proteins, green indicates down-regulated proteins.

In this review, we include the output of the interaction between rice (resistance or susceptible) and *M. oryzae* (compatible or incompatible race) determined by plant immunity and PAMP molecular analyses. Here, 18 papers (published from 2003 to 2019) on rice proteome responses to *M. oryzae* and related molecules that cause PAMPs or R protein-induced resistance—as well as other type of resistance against rice blast disease—are reviewed (**Table 3**). Moreover, the majority of these studies utilized a 2-DE-based approach, with fewer reports utilizing the shotgun proteomics approach. The major finding provided by the proteomics studies in rice−*M. oryzae* pathosystem and PAMPs treatment are illustrated in **Figure 2**.

**Table 3:** A list of proteomic studies focused on responses to *M. oryzae* infection and its derived molecular treatment in rice.

Resistance	Plant	Treatment	Methods	Protein section	Major findings	References
*M. oryzae* infection	Rice suspension-cultured rice cells	24 and 48 hpi M. oryzae (KJ401), JA, SA, or H_2_O_2_	2-DE, *N*-terminal or internal amino acid sequencing	Total protein	12 proteins were induced, including OsPR-10, isoflavone reductase-like protein, β-glucosidase, and putative receptor-like protein kinase, PBZ1, SalT.	(Kim et al., 2003)
	Rice leaves (*Oryzae sativa* cv. Jinheung)	24, 48, and 72 hpi *M. oryzae* (incompatible [KJ401] and compatible [KJ301] races)	2-DE, MALDI-TOF MS	Total proteins	Eight proteins were induced, including two RLKs, two β -1.3-glucanases (Glu1, Glu2), TLP, POX 22.3, PBZ1, and OsPR-10.	(Kim et al., 2004)
	Rice leaves (Nipponbare)	24 and 72 hpi *M. oryzae* strains Guy11 and JS153	iTRAQ labeling	Total protein	634 proteins were identified. Proteins responding to oxidative stress and biotic stress were enriched.	(Lin et al., 2018)
	Rice leaves (Nipponbare as WT and ABA-insensitive mutant Osabi3)	24 hpi *M. oryzae*	iTRAQ labeling	PM proteins	484 of 3,906 identified proteins were significantly modulated. ABA and CK signaling were sequentially activated after *M. oryzae* infection in rice.	(Cao et al., 2016)
	Rice seedling (Oryza sativa L) Moroberekan cultivar, a blast resistant rice cultivar	0, 2, 6, 12, 24, 48, 72, 96, 120, 144, and 168 hpi *M. oryzae*	2-DE and LC-MS/MS	Nuclear proteome	A total of 140 immune-responsive proteins (IRPs) were identified associated with nuclear reorganization, cell division, energy production/deprivation, signaling and gene regulation	(Narula et al., 2018)
	Rice suspension (Oryza sativa L. cv Jinheung) suspension-cultured cells	24 hpi *M. grisea* (KJ401) and its elicitor	2-DE, MALDI-TOF-MS and mLC-ESI-MS/MS	Apoplast localized proteins	21 proteins were identified in response to *M. grisea* and/or elicitor. Most of the assigned proteins were involved in defense such as nine chitinases, two germin A/oxalate oxidases, five DUF 26 secretory proteins, and β-expansin.	(Kim et al., 2009)
	Rice leaves (Japonica cv Kakehashi)	12 and 72 hpi *M. oryzae* Ken54-20 (incompatible) and Ina186-137 (compatible)	2-DE and LC–MS/MS	Apoplast localized proteins.	Three DUF26 domain proteins and a Magnaporthe Cyclophilin were identified	(Shenton et al., 2012)
	Rice seedlings (Oryza sativa L. cv Jinheung)	72 hpi *M. oryzae* (incompatible [KJ401] and compatible [KJ301] races)	2-DE, MudPIT MALDI-TOF-MS, and nESI-LC–MS/MS	Apoplastic secretome	Of 732 identified proteins, 291 and 441 proteins were derived from rice and *M. oryzae*, respectively. Among these, rice secretes proteins related to stress response, ROS and energy metabolism; *M. oryzae* secretes proteins involved in metabolism and cell wall hydrolyses	(Kim et al., 2013)
PAMP induced resistance	Rice leaves (japonica cv. Nipponbare)	24 hpt MoHrip2	2-DE MALDI-TOF/TOF	Total protein	17 differentially expressed proteins were involved in defense-related transcriptional factors, signal transduction-related proteins, ROS production, programmed cell death (PCD), defense-related proteins, and photosynthesis and energy-related proteins	(Khan et al., 2016)
	Rice leaves (*Oryzae sativa* cv. Dongjin)	24 hpt MSP1 and flg22	Label-free quantitative	Total protein	433 of 4,167 identified proteins were significantly modulated. Proteins related to primary, secondary, and lipid metabolism were decreased, while proteins associated with the stress response, PTM, and signaling were increased in abundance	(Meng et al., 2019)
	Rice leaves (*Oryzae sativa* cv. Dongjin)	24 hpt and 3 hpt MSP1 for proteome and phosphoproteome respectively	TMT-based and Label-free quantitative	Cytosolic and plasma membrane proteome	6691 proteins and 1906 phosphoproteins were identified which collectively showed activation of proteins related to the proteolysis, jasmonic acid biosynthesis, redox metabolism, and MAP kinase signaling pathways in response to MSP1 treatment	(Gupta et al., 2019)
	Rice seedlings (Nipponbare)	3 hpt chitin and flg22	Label-free quantitative	Ubiquitin-related proteins	The ubiquitination levels of many proteins involved in the ubiquitination system, protein transportation, ligand recognition, membrane trafficking, and redox reactions were significantly changed in response to the elicitor treatments.	(Chen et al., 2018)
R protein-mediated resistance	RIL260 rice strain carrying the *Pi-5* resistance gene, susceptible mutants M5465 and M7023	0, 24, and 48 hpi *M. oryzae*	2-DE, MALDI-TOF MS	Total proteins	Eight proteins were differentially modulated in the mutant strain, including three downregulated proteins (triosephosphate isomerase, 2,3-bisphosphoglycerate-independent phosphoglycerate mutase) and four upregulated proteins (fructokinase I, a GST, an atpB of chloroplast ATP synthase, an aminopeptidase N)	(Ryu et al., 2009)
	Rice leaf sheaths (resistant cultivar, ZTR, which contains *Pi-zt* gene, and susceptible rice plants, ZTS)	40 hpi *M. oryzae* and ABA treatment	2DE, MALDITOF MS	Total proteins	13 DMPs were identified. Induction of the thaumatin-like protein after the fungal inoculation is associated with the expression of WPSR in the susceptible rice plant	(Koga et al., 2012)
	Rice leaves (Oryza sativa L. Nipponbare [NPB] and transgenic NPB [NPB-Piz-t] harboring *Piz-t* gene)	24, 48, and 72 hpi *M. oryzae* isolates KJ201 (avirulent to Piz-t) and RB22 (virulent to Piz-t)	iTRAQ labeling	Total protein	DEPs, in comparisons between incompatible and compatible interactions, contained a number of proteins, including PR proteins, hormonal regulation-related proteins, defense and stress response-related proteins, RLK, and cytochrome P450.	(Tian et al., 2018)
	Rice leaves (Oryza sativa indica C101LAC and CO9, among which, C101LAC contains the resistance gene Pi-1 in the CO39 background	8, 12, and 24 hpi *M. oryzae*	2-DE, MALDI-TOF/TOF MS, and nanoLC-MS/MS	Phosphoproteins	53 phosphoproteins were identified. defense-related proteins, signaling-related proteins, microtubule-associated proteins, energy-related enzymes, and amino acid synthesis-related proteins differ in compatible and incompatible interactions.	(Li et al., 2015)
Other type of resistance	Rice leaves (Background line CO39, resistant line C101LAC containing Pi-1)	12, 24 and 48 hpt SA treatment	2DE, MALDI-TOF/TOF MS	Total proteins	Among 36 DMPs, proteins involved in defense, signal transduction and antioxidative enzymes were induced in resistant line except three antioxidative enzymes, indicating resistant rice cultivar might possess a more sensitive SA signaling system or effective pathway than susceptible cultivar.	(Li et al., 2012)
	Rice leaves (CO39 susceptible to M. oryzae)	4 days after *M. oryzae* inoculation and/or Si treatments	2-DE and LC-MS/MS	Total proteins	61 proteins were identified. Si-regulated proteins were involved in energy/metabolism, photosynthesis, redox homeostasis, protein synthesis, transcription, and pathogen response.	(Liu et al., 2014a)

### *M. oryzae* Responsive Proteome

To study the rice proteome in response to *M. oryzae* infection, changes in apoplastic (Kim et al., 2009; Shenton et al., 2012; Kim et al., 2013), PM (Cao et al., 2016), nuclear (Narula et al., 2018), and total proteome were investigated (Kim et al., 2003; Kim et al., 2004; Lin et al., 2018). Most of these studies compared rice infected with a compatible and incompatible blast fungus, revealing the proteome response of rice to different types of infection strategies.

At the very beginning, the abundance of proteins involved in cell signaling is altered in plants responding to pathogen attack, including pathogen perception and signal transduction. Early perception of the pathogen occurs in the apoplast by the apoplastic and PM-localized proteins (Gupta et al., 2015a). The apoplast serves as an interface for the exchange of nutrients and signals between plant cells and the surrounding environment. Several proteomic studies in rice leaves or suspension-cultured cells treated with the rice blast fungus (compatible and incompatible race) have highlighted the regulation of apoplastic proteins involved in pathogen recognition (Kim et al., 2009; Shenton et al., 2012; Kim et al., 2013).

Domain unknown function 26 (DUF26) containing proteins, which are usually found in serine/threonine kinases are associated with the plant receptor-like kinase. Several studies reported that DUF26 proteins were up regulated upon *M. oryzae* or elicitor treatment. In the secretome of rice suspension-cultured cells inoculated with *M. oryzae* and rice seedling inoculated with *M. oryzae*, five and six DUF26 secretory proteins accumulated earlier in incompatible interactions than in compatible ones, respectively (Kim et al., 2009; Kim et al., 2013). The earliest accumulation of DUF26 proteins was reported in the rice apoplast 12 h after inoculation with *M. oryzae* (Shenton et al., 2012). Among them, one common DUF26 (LOC_Os04g56430.1) was observed to be induced by *M. oryzae* (compatible and incompatible race) inoculation, MSP1 treatment, JA treatment and wounding (Kim et al., 2003; Kim et al., 2004; Gupta et al., 2019; Meng et al., 2019), suggesting that this protein may mediate defense responses in rice through the JA pathway.

Moreover, a PM proteomic study of rice leaves challenged by *M. oryzae* reported that two NB-LRR R proteins and several receptor-like kinases (RLKs) were up-regulated at 48 hpi but not at 24 hpi (Cao et al., 2016), suggesting that NB-LRR R proteins-mediated ETI during rice-*M. oryzae* interaction is activated at around 48 hpi. Pathogen infection also affects the cellular transport and membrane properties for initiation of defense signaling at the PM. For instance, an accumulation of PM transporters, such as vesicle trafficking-related proteins, has been observed in the PM proteomic study (Cao et al., 2016). Altogether, these proteins may play a role in *M. oryzae* perception and signal transduction in rice.

Plant defense responses comprise the reinforcement of cell walls, the production of phytoalexins, and the synthesis of defense-related proteins. Defense-related proteins refer to proteins that are components of the signal transduction pathways leading to defense responses of the host after pathogen perception (Bowles, 1990). As well-known defense-related proteins, pathogenesis-related (PR) proteins accumulate during pathological conditions and related abiotic stress, possessing antimicrobial properties (van Loon et al., 2006). PR proteins have been classified into 17 families according to their structural and functional properties, and they are involved in a wide range of functions, including cell wall reinforcement, signal transduction, and antimicrobial activity (van Loon et al., 2006). For instance, chitinases and glucanases, involved in the degradation of fungal and oomycete cell walls, are induced by pathogenic infection and abiotic factors. Among the proteomic studies of the rice−*M. oryzae* pathosystem, several PR proteins have been found to be associated with plant defense. In the secretome studies, 10 PR-5s, nine chitinases, and six PR-1s were induced by the rice blast fungus (compatible and incompatible race) in rice leaves and suspension-cultured cells (Kim et al., 2009; Kim et al., 2013). Moreover, in suspension-cultured rice cells and rice leaves, PBZ1, OsPR-10, SalT, Glu1, Glu2, and TLP proteins were up-regulated by *M. oryzae* infection (compatible and incompatible race; Kim et al., 2003; Kim et al., 2004).

Disorders in cellular metabolism under pathogen attack lead to an enhanced risk of oxidative damage. The long-held conception of ROS as harmful has now changed, with compelling evidence gathered over the years showing that ROS acts as signaling molecules to regulate plant processes such as cell death and pathogen defense. Enhanced production of apoplastic ROS during a pathogen attack is involved in strengthening of the cell wall and function as local and systemic signal molecules associated with the activation of antimicrobial defenses, which is related to disease resistance (Waszczak et al., 2018). Moreover, transient and instant production of apoplastic ROS, called ROS burst, is a hallmark of PTI defense signaling (Torres et al., 2006). Apoplastic ROS production results from the accumulation of ROS-producing enzymes composed of apoplastic peroxidases, polyamine oxidases (PAOs), and PM-localized NADPH oxidases (respiratory burst oxidase homologs, RBOHs; Waszczak et al., 2018). At the proteomic level, abundance of 15 apoplastic peroxidases and two germin A/oxalate oxidases were highly increased in the secretome studies during rice−*M. oryzae* interaction (Kim et al., 2009; Kim et al., 2013). Moreover, in the PM proteomics study, an accumulation of a rice NADPH oxidase encoded by *Osrboh8* was observed at 48 hpi in rice leaf samples inoculated with *M. oryzae* (Cao et al., 2016). Thus, apoplastic peroxidases and NADPH oxidases play a key role in regulating apoplastic ROS, in association with apoplastic ROS-mediated disease resistance.

The concentration and longevity of ROS are determined by the composition and availability of antioxidant systems, while the compartmentalization of ROS production and scavenging shapes the function of ROS in the plant (Waszczak et al., 2018). Numerous proteomics studies have reported that enzymes involved in ROS production, such as superoxide dismutase (SOD) and class III peroxidases, as well as enzymes associated with the detoxification of ROS including catalase (CAT), ascorbate peroxidase (APX), glutathione S-transferase (GST) and dehydroascorbate peroxidase, accumulate in plants invaded by pathogens (O’Brien et al., 2012; Kim et al., 2014).

Among these, class III peroxidases can either produce H_2_O_2_ in oxidase cycle or scavenge H_2_O_2_ to H_2_O and O_2_ through peroxidase activity (Bolwell et al., 1999; Waszczak et al., 2018); class III peroxidases were reported to be required for pathogen resistance in Arabidopsis (Bindschedler et al., 2006). In the secretome study, 20 peroxidases were highly abundant in rice following *M. oryzae* infection (compatible and incompatible race; Kim et al., 2013). Furthermore, an increased abundance of several ROS-scavenging enzymes were observed in incompatible interactions between rice and *M. oryzae*, such as OsPRX59 and OsPRX62 in rice inoculated with *M. oryzae* avirulent strain JS153 (Lin et al., 2018). In the nuclear proteome study, a continuous increase of APX and thioredoxin peroxidase, mixed regulation of GST and thioredoxin, and the continuous decrease of DHAR were observed in rice (resistant cultivar) inoculated by *M. oryzae* (Narula et al., 2018).

Pathogen infection also led to an enhanced risk of protein damage due to imbalances in cellular homeostasis and secretion of proteases by the pathogens (Wang et al., 2017). To prevent accumulation of unwanted proteins, and to facilitate the refolding of denatured proteins, plant HSPs, a class of molecular chaperones, become enriched upon pathogen infection. OsHSP81 was induced by both virulent and avirulent *M. oryzae* strains in 72 hpi rice samples, which functions to induce both ROS production and callose deposition (Lin et al., 2018). Moreover, induction of one 60 kDa chaperonin and four 70 kDa heat shock cognate proteins in the early stages, and repression of one HSP17.5 and two HSP 70, were observed in a rice nuclear proteome study after being challenged with an incompatible race of *M. oryzae* (Narula et al., 2018).

In this nuclear proteome study, alteration of nuclear reorganization-related proteins, including induction of two nuclear actin molecules and down-regulation of actin depolymerizing factor, were reported in rice in response to exposure to the incompatible strain of *M. oryzae* (Narula et al., 2018). Further, changes in the abundance of DNA- and RNA-binding proteins, such as up-regulation of Alba and two DNA/RNA-binding proteins, as well as down-regulation of two nucleic acid-binding proteins, were observed in rice samples infected by *M. oryzae*. Additionally, transcription factors, including zinc finger, RING-type domain-containing protein, and NAC transcription factor were up-regulated after fungal attack. Early repression—and later induction—of FK506-binding protein (FKBP), as well as repression of enoyl-acyl-carrier-protein reductase (NADH) and ferredoxin-NADP reductase were found in response to *M. oryzae* invasion. Moreover, glycolytic enzyme, increase of TPI and protein phosphatase 2C, dysregulation of FBA, and repression of GAPDH were observed in response to *M. oryzae* infection, modulating glycolytic flux, cell proliferation, and chromatin modification in attempts to resist fungal attack (Narula et al., 2018).

Phytohormones are central regulators of plant defense. SA, JA, and ET are three main hormones that play crucial roles in plant immunity. There is a dichotomy in plants, such, that SA is involved in the regulation of immunity against biotrophic pathogens, whereas JA and ET are considered to be regulators of resistance to necrotrophic and insect pests (Browse, 2009; Pieterse et al., 2012). However, rice plants infected by *M. oryzae* and *Xanthomonas oryzae* pv *oryzae* do not show any elevation in SA levely, whereas exogenous SA does, indeed, induce a defense response in rice plants (Silverman et al., 1995).

In the case of JA, an increase of the endogenous jasmonate isoleucine (JA-Ile), a bioactive form of JA, has been found in response to *M. oryzae* infection. JA-Ile is synthesized by the activity of two jasmonate synthases, including OsJAR1 and OsJAR2, and it was shown that *M. oryzae*-induced JA-Ile production is mediated, in particular, by OsJAR1 (Wakuta et al., 2011; Zhang et al., 2018). Additionally, compelling evidence has been gathered over the years showing activation of JA signaling in response to biotrophic, hemibiotrophic, and necrotrophic pathogens (Mei et al., 2006; Jiang et al., 2010; Yamada et al., 2012).

SA and JA are major defense-related phytohormones, and function antagonistically to one another in dicot plants in order to regulate plant defense signaling (Loake and Grant, 2007; Betsuyaku et al., 2018). However, this cross-talk between SA and JA signaling in monocot plants remains ill-defined. Although a decrease in endogenous SA concentration was observed upon exogenous application of JA in rice leaves, an increase in some of the common defense marker genes, such as PR1b, has been observed in response to exogenous treatment of both JA and SA (Tamaoki et al., 2013). Moreover, a two-fold increase in OsWRKY45, a key component of the SA signaling cascade in rice, was observed in response to JA treatment. Altogether, these results suggest that both JA and SA coordinate defense signaling in rice. Owing to the high endogenous levels of SA in rice leaves (>1 µg/g fresh weight), SA has been proposed to contribute to the basal resistance under normal conditions, and, after activation of JA signaling, SA levels decrease, resulting in suppression of SA signaling and activation of common defense signaling by JA (Tamaoki et al., 2013).

Although no direct indication of suppression of SA signaling by JA has been observed to date, there is clear evidence that ABA antagonizes SA signaling to suppress an immune response in rice (Jiang et al., 2010) and Arabidopsis (Berens et al., 2019). A PM proteomics study reported that ABA signaling was activated at an early stage of *M. oryzae* infection, suppressing the plant immune response for initial invasion, while cytokinin (CK) signaling was activated at a later stage of the pathogen infection (Cao et al., 2016).

### PAMP Responsive Proteome Changes in Rice

PAMPs in the rice−*M. oryzae* pathosystem include chitin, MSP1, and MoHrip1. Overexpression of MSP1 in rice confers broad-spectrum resistance against *M. oryzae* and *Xanthomonas oryzae* pv *oryzae* (Hong et al., 2017). Proteomic studies of rice treated with PAMPs provides a new insight into the mechanism of blast resistance induced by PAMPs. Here, we reviewed four recently published proteome studies of rice treated with chitin, MSP1, or MoHrip2 (Khan et al., 2016; Chen et al., 2018; Meng et al., 2018a; Gupta et al., 2019; Meng et al., 2019). Among these, three studies utilized gel-free shotgun proteomics, including one ubiquitome study in rice treated with chitin and flg22.

At the stage of PAMP detection, several RLKs accumulated after PAMP treatment, including RLCK109 and BIP128 in rice leaves treated with MSP1 (Meng et al., 2018b; Meng et al., 2019). In the ubiquitome study, ubiquitination levels of some RLKs, such as OsRLCK103, OsRLCK107, OsSERK2, and a BAK1-like protein, were up-regulated by chitin, while several receptors or protein kinases, including OsRLCK64, OsRPK1, OsNek6, OSK22, OsRLK1, OsCrRLK1L3, OsBURP16, SnRK1A, and OsPGK4, showed down-regulated ubiquitination levels in response to chitin treatment (Chen et al., 2018). These results allow us to gain further understanding of how ubiquitination of PM receptor kinases contributes to plant immunity.

Moreover, an increased abundance of several PR proteins was observed in rice treated by *M. oryzae* elicitors (Gupta et al., 2019). Examples include PBZ1, OsPR-10, TLP, OsChib3a, chitinase RCB4, and OsDR8 in rice leaves treated with MSP1 (Meng et al., 2019), and PR-5 and PR-10 in rice leaves treated with MoHrip2 (Khan et al., 2016). Other defense-related proteins were also elevated by elicitor treatment, such as germin-like proteins (GLPs) and cinnamyl alcohol dehydrogenases (CADs). Of these, GLPs were reported to confer disease resistance in a variety of plants, including rice, barley, and wheat (Manosalva et al., 2009), and CADs are involved in lignin biosynthesis and the phenylpropanoid pathway (Sibout et al., 2005).

The phenylpropanoid pathway is associated with the synthesis of antioxidants, contributing to detoxification, or which act as signaling molecules to mediate defense responses (Dixon et al., 2002). Three CADs (CAD3, CAD8B, and CAD8C) and six GLPs (GLP1-1, GLP2-4, GLP3-2, GLP8-10, GLP8-12, and GLP3-7) were found to accumulate in rice leaves treated with MSP1 (Meng et al., 2019). Moreover, ubiquitination levels of many enzymes in the phenylpropanoid pathway, including OsPAL1, OsPAL2, OsPAL7, OsPAL5, Os4CL3, OsC4HL, OsCHS1, OsCOMT1, OsCAD2, XDH1, UDPGT, and OsF5H, were significantly increased in response to chitin treatment (Chen et al., 2018).

Accumulation of ROS occurs as a means of a defensive response when plants are challenged by pathogens, while enzymes involved in ROS production and scavenging, such as PM-localized NADPH oxidases (RBOHs), class III peroxidases, APX, and GST, were enriched to balance cellular redox homeostasis (Gupta et al., 2019). For instance, OsGSTU46, OsGSTF14, and OsGSTZ3, as well as OsPOX22.3, OsPOX5.1, prx59, prx65, prx78, and prx122 in rice treated with MSP1 were up-regulated (Meng et al., 2018a). Moreover, ubiquitination levels of OsRbohA and OsRbcL1 were increased in rice treated with chitin, while ubiquitination levels of GAPDH, OsAPX1,OsAPX2, OsTRXh2, OsGPX1, and OsGSTT1 were decreased in response to chitin treatment (Chen et al., 2018).

HSPs are induced by different stress conditions and play an important role in maintaining protein function and structure. Here, HSPs were elevated in rice treated with elicitors. For instance, three HSPs, including DnaK family protein and ERD1 (early-responsive to dehydration 1), were induced in response to MSP1 treatments (Meng et al., 2018b). Moreover, ubiquitination levels of OsMed37_1, OsHSP71.1, and OsHSP82A were elevated in response to chitin treatment (Chen et al., 2018). Additionally, hormone signaling-related protein expression changed in response to elicitors treatments. In ubiquitome study, five proteins (OsBRI1, OsBRD2, OsSERK2, BIP102, and BIP106) involved in the brassinosteroid signaling pathway showed significantly increased ubiquitination levels in response to chitin treatment, while ubiquitination levels of GID2 involved in the GA signaling pathway were decreased (Chen et al., 2018).

### R Protein-Mediated Proteome

Currently, more than 100 genes that confer rice blast resistance have been identified in rice, such as *Pi-1*, *Pi-5*, and *Pi-z* *^t^*, resulting in the development of rice blast resistance cultivars harboring R genes. Here, we reviewed three comparative proteome studies of rice cultivars with R genes (*Pi-1*, *Pi-5*, and *Pi-z* *^t^*) revealed important proteomic changes in response to *M. oryzae*.

In the comparative proteome study of rice strains carrying the *Pi5* R gene with susceptible mutants against challenge of *M. oryzae*, eight proteins were differentially regulated between resistant and susceptible plants. Among them, an abundance of three proteins were found to be increased in resistance cultivar, including a triosephosphate isomerase, a 2,3-bisphosphoglycerate-independent phosphoglycerate mutase, and an unknown protein, while five downregulated proteins included a fructokinase I, a GST, an atpB of chloroplast ATP synthase, an aminopeptidase N, and an unidentified protein (Ryu et al., 2009). A comparative proteome study was performed between two incompatible interactions (*M. oryzae* vs. ZTS rice leaf sheaths expressing whole plant-specific resistance [WPSR] and resistant rice ZTR (carrying *Pi-zt*) and one compatible interaction (*M. oryzae* vs. ZTS rice leaf sheaths in which WPSR was suppressed by ABA).

A total of 13 proteins, including PR-2, PR-5, and PR-10, were differentially modulated between the compatible and incompatible interactions (Koga et al., 2012). Loss-of-function of PR-5 significantly suppressed WPSR, suggesting that induction of PR-5 by *M. oryzae* is associated with the expression of WPSR in the susceptible rice plant (Koga et al., 2012). Additionally, another proteomics study of *Pi-z* *^t^* transgenic rice line NPB-Piz-t and wild type rice (NPB infected by an avirulent *M. oryzae* isolate KJ201 and a virulent isolate RB22) was carried out by iTRAQ analysis. In this study, five PRs, including OsCHIT7/endochitinase, BBTI-4/RBBI3–1, OsCHIB1, Gns12, and OsLTP2, were significantly increased in the incompatible interaction (KJ201-Piz-t) relative to the compatible one (KJ201-NPB or RB22-Piz-t). Moreover, an RLK was observed to be induced by *M. oryzae* inoculation in the NPB and NPB-Piz-t rice lines. In addition to PRs and RLKs, a putative bowman birk trypsin inhibitor, which might be involved in AvrPiz-t/Piz-t networks, was induced by *M. oryzae* infection in both compatible and incompatible interactions (Tian et al., 2018).

The phosphorylation and dephosphorylation of proteins, catalyzed by kinases and phosphatases, respectively, affects protein configuration, ultimately resulting in modified protein with novel enzymatic functions, substrate specificity, structural stability, or intracellular localization (Kline-Jonakin et al., 2011). Phosphoproteomic studies of rice leaf samples from resistant (i.g., carrying the resistance gene *Pi1*) and susceptible rice cultivars invaded by *M. oryzae* led to the identification of 53 regulated phosphoproteins. Among them, many ROS-related enzymes were differentially modulated by phosphorylation in both cultivars, including enzymes implicated in ROS accumulation (i.g., Cpn60α and Cpn60β) and ROS detoxification (i.e., cysteine synthase and ROS-scavenging enzymes). Of these, all ROS-scavenging enzymes decreased in the susceptible or resistant strain or in both the cultivars after *M. oryzae* inoculation, suggesting phosphorylation in response to pathogen attack helps generate an oxidative burst in both the cultivars. Moreover, higher H_2_O_2_ accumulation was observed in the incompatible interaction relative to the compatible interaction at all time points within 24 h after inoculation. Phosphorylated cinnamoyl-CoA reductase (CCR), a key enzyme in lignin biosynthesis, was found to be down-regulated in susceptible cultivars at 8 h after inoculation. Additionally, phosphorylated WRKY11 was up-regulated in the resistant cultivar C101LAC at 12 h post-inoculation, while phosphorylated CHP-rich zinc finger protein-like was down-regulated in the susceptible rice (Li et al., 2015).

### SA/Si-Responsive Proteome

Plants have a variety of inducible defense mechanisms that can be triggered by different biotic and abiotic stimuli. Due to the central importance of SA and JA in plant immunity, proteomic analysis of rice leaves in response to exogenous SA treatment has also been conducted. Moreover, Silicon (Si) can significantly enhance plant resistance against various pathogens. Here, we summarized two proteome studies of rice treated with SA and Si.

In the proteomics study of rice blast resistant cultivar (carrying the *Pi1* gene) and susceptible cultivar treated with SA, 36 proteins were differentially regulated. Among them, selenium-binding protein (SBP) and phenylalanine ammonia-lyase (PAL) were induced at 12 h, CAD and endo-1,3-1,4-ß-glucanase were induced at 24 h, and ß-1,3-glucanase was induced at 48 h post-SA treatment, with PAL and ß-1,3-glucanase only induced in resistant cultivars. Antioxidant enzymes were significantly decreased in response to SA treatment, such as APX, Prx, and SOD1, suggesting SA may cause an oxidative burst to help prevent plants from succumbing to fungal infection. Nucleoside diphosphate kinase 1 (NDPK1), which is involved in signaling transduction, was significantly increased at 24 h after SA treatment in resistant cultivars. Chaperonin α subunit and protein disulfide isomerase (PDI) were induced by SA in both cultivars at 24 hours post-treatment (hpt), while PDI and chaperonin 21 were only found to be induced in susceptible cultivars at 48 hpt. Proteomics study of SA-induced rice resistance to *M. oryzae* reveals that resistant rice cultivars might possess a more sensitive SA signaling system than susceptible cultivars (Li et al., 2012).

A proteomic study of rice blast susceptible cultivar challenged by *M. oryzae* inoculation and/or Si application showed that 43 proteins were differentially modulated by the addition of Si to the *M. oryzae*-inoculated rice. Three antioxidant enzymes, including cytosolic L-ascorbate peroxidase 1, dehydroascorbate reductase, and superoxide dismutase, were significantly suppressed by *M. oryzae* inoculation, but increased by Si application. Moreover, elongation factor Tu was increased by *M. oryzae* infection alone but decreased by Si application. A glycine-rich RNA-binding protein was down-regulated by *M. oryzae* but up-regulated by Si application. Moreover, Si application altered PR10 and GSTU6, after which *M. oryzae* induced PR10 but suppressed GUST6 (Liu et al., 2014a).

## Conclusions and Perspectives

Plant responses to pathogens involves proteomic reorganization, such as the synthesis of cell wall-modifying enzymes for the reinforcement of the PM, and defense-related proteins for the enhancement of immune and defense responses. Comparative proteomics studies of incompatible and compatible interactions between rice and *M. oryzae* provides an overview of important protein changes involved in rice resistance against *M. oryzae*, including energy metabolism, pathogen recognition, defense-related proteins, hormone signaling, ROS, and redox homeostasis. With advancements in proteomic technologies, we can explore the defense-responsive proteins and corresponding protein PTM sites in a high-throughput and time-efficient manner, followed by their application in the breeding of broad-spectrum resistant varieties.

In the case of rice−*M. oryzae* interactions, few gel-free proteomics studies have been performed to date, resulting in the generation of limited proteomic data for breeding use which may, in turn, contribute to inaccurate predictions. For rice−*M. oryzae* interactions, the apoplastic space represents an important contact area between rice and *M. oryzae*. Proteomic analysis of the apoplast in recent years has found the initial biochemical responses involved in pathogen recognition and early defense responses. Thus, more apoplastic proteomics studies are required for a more comprehensive and nuanced understanding of the key processes of pathogen infection that determine the fate of plant−pathogen interactions.

## Author Contributions

QM, RG, and STK wrote the manuscript. CWM, SWK, YW, BIJ, Y-JK, J-SJ, GKA, and RR critically reviewed and proofread the manuscript.

## Funding

This work was supported by grants from the Basic Science Research Program through the National Research Foundation of Korea (NRF) funded by the Ministry of Education, Science and Technology (NRF-2018R1A4A1025158) provided by STK. RG was supported by the Ramalingaswami-re-entry fellowship (BT/HRD/35/02/2006) from the Department of Biotechnology, Ministry of Science and Technology, Government of India.

## Conflict of Interest

The authors declare that the research was conducted in the absence of any commercial or financial relationships that could be construed as a potential conflict of interest.
